# Differential isoform expression and alternative splicing in sex determination in mice

**DOI:** 10.1186/s12864-019-5572-x

**Published:** 2019-03-12

**Authors:** Benjamín Planells, Isabel Gómez-Redondo, Eva Pericuesta, Patrick Lonergan, Alfonso Gutiérrez-Adán

**Affiliations:** 1 Departamento de Reproducción Animal, Instituto Nacional de Investigación y Tecnología Agraria y Alimentaria, Madrid, Spain; 2 School of Agriculture and Food Science, University College Dublin, Dublin, Ireland

**Keywords:** Sex determination, Gonads, RNA-seq, Transcriptomics

## Abstract

**Background:**

Alternative splicing (AS) may play an important role in gonadal sex determination (GSD) in mammals. The present study was designed to identify differentially expressed isoforms and AS modifications accompanying GSD in mice.

**Results:**

Using deep RNA-sequencing, we performed a transcriptional analysis of XX and XY gonads during sex determination on embryonic days 11 (E11) and 12 (E12). Analysis of differentially expressed genes (DEG) identified hundreds of genes related to GSD and early sex differentiation that may represent good candidates for sex reversal. Expression at time point E11 in males was significantly enriched in RNA splicing and mRNA processing Gene Ontology terms. Differentially expressed isoform analysis identified hundreds of specific isoforms related to GSD, many of which showed no differences in the DEG analysis. Hundreds of AS events were identified as modified at E11 and E12. Female E11 gonads featured sex-biased upregulation of intron retention (in genes related to regulation of transcription, protein phosphorylation, protein transport and mRNA splicing) and exon skipping (in genes related to chromatin repression) suggesting AS as a post-transcription mechanism that controls sex determination of the bipotential fetal gonad.

**Conclusion:**

Our data suggests an important role of splicing regulatory mechanisms for sex determination in mice.

**Electronic supplementary material:**

The online version of this article (10.1186/s12864-019-5572-x) contains supplementary material, which is available to authorized users.

## Background

The majority of eukaryote genes have multiple transcriptional isoforms. Originating from the same locus, mRNA isoforms are molecules of different exon composition and length, which may code for different forms of the corresponding protein. These isoforms may be produced from different transcriptional starting sites and terminated at different polyadenylation sites, or may be a consequence of alternative splicing (AS) [1]. AS is a ubiquitous regulatory mechanism of gene expression that has functions in developmental processes, regulating tissue and organ development and disease [2]. AS may affect mRNA localization, stability, translation, or may change the reading frame, resulting in different protein isoforms with diverse functions and/or localizations [3]. Sequences of isoforms found on the basis of experimental evidence are deposited in public databases and are available through the Ensembl genome browser (www.ensembl.org). However, there are many isoforms produced by AS that are not documented, but could be functionally important [4]. Isoform changes may be masked by gene-level measurements. Thus, the examination of developmental dynamics, estimating isoform expression and new AS events offers improved resolution over gene expression.

Gonadal sex determination (GSD) is the primary event in mammalian sexual development during which bipotential gonads differentiate into either testes (in males) or ovaries (in females). This decision is made after *Sry* expression in the bi-potential XY gonad [5] which initiates the upregulation of *Sox9* expression which, in turn, stimulates *Fgf9* expression. Next, both *Fgf9* and *Sox9* act together in a positive feedback loop, which is thought to suppress *Wnt4*, establishing the testis-specific pathway. In XX individuals lacking *Sry*, specific female genes such as *Rspo1*, *Wnt4*, *Ctnnb1* and *Foxl2* are expressed at high levels, suppressing *Sox9*/*Fgf9* positive feedback and initiating ovarian differentiation. Most of the main genes related to sex determination in mammals are known. However, there is a lack of information about RNA isoforms and AS variants involved in germline development. These mechanisms are responsible for sex determination in some insects including flies, honeybees, and wasps [6], and in some reptiles [7]. Moreover, certain transcription factors important for sex determination in mammals, such as *Wt1*, *Sry* and *Sox6*, control mRNA splicing [8, 9]. Further, *Sox9* has been shown to modulate either transcription or splicing of distinct sets of targets in colon tumour cells [10]. In addition, during sex differentiation in mice, *Sox9* not only regulates transcription of its target genes directly, but also influences their RNA splicing [9]. Analysis of these AS events related to sexually dimorphic transcription programmes in developing fetal gonads is crucial also for understanding the etiology of human disorders of sexual development (DSD), many of which remain unexplained.

The ability of *Sry* to secure testis fate is limited to a time window of approximately 6 h after the normal onset of *Sry* expression, and this is crucial to switch from female to male signalling in the developing gonads. Thus, delayed *Sry* induction is not capable of switching these signals [11]. For our analysis, we selected time-points before and after peak *Sry* expression on embryonic day 11.5 (E11.5) in order to characterize expression in the bi-potential male and female gonads at E11, and early sex differentiation in male and female gonads at E12. We explore the genome-wide transcriptome landscape to identify gene-, isoform-, and AS-level expression features related to sex determination and early differentiation in mice. Hundreds of new genes related to GSD and early differentiation were detected. These genes are potentially involved in disorders of sexual development. In addition, hundreds of candidate RNA isoforms and AS variants, which potentially regulate GSD and early differentiation, were also identified.

## Results

### RNA-seq analysis and sex-dependent differential gene expression before and after the *Sry* expression peak in mouse gonads

**Fig. 1:**
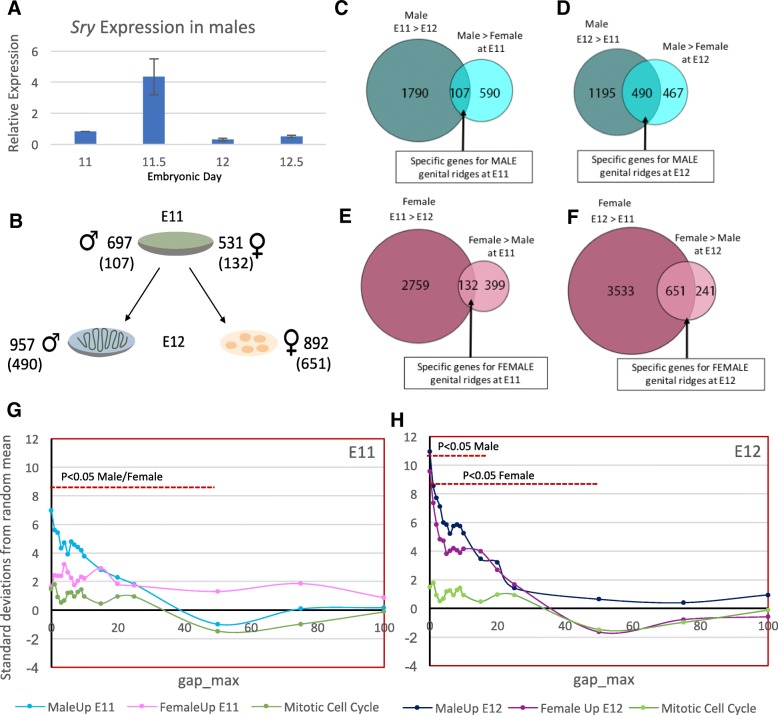
Differentially expressed genes (DEG) in male and female gonads at E11 and E12 and analysis of gene clustering. (**a**) Quantitative PCR analysis of *Sry* expression in male embryonic day 11 and 12.5 (E11-E12.5) gonads. Biological triplicate results are presented as mean ± SEM. Bars with different superscripts differ significantly (ANOVA, *P* < 0.05). (**b**) Diagram showing the genes upregulated in E11 and E12 male and female gonads. In brackets is the number of specific genes as observed in graphs C to F. (**c-f**) Venn diagrams showing DEGs in E11 and E12 male and female gonads. Subset of genes represent specific upregulated genes in males at E11 (**c**) and E12 (**d**), and in females at E11 (**e**) and E12 (**f**). Analysis with Cluster Locator [15] of clustering of genes upregulated in E11 (**g**) and E12 (**h**) male and female gonads

To identify the initial molecular changes associated with GSD, we first confirmed by qPCR that peak *Sry* expression in gonads occurs at time point E11.5 (Fig. 1A). We then selected two different time points (E11 and E12, before and after the *Sry* peak, respectively) for RNA deep sequencing (Fig. 1B). We pooled three pairs of genital ridges from three different XX or XY individuals at the two time points to minimize the effect of biological variability and performed RNA-seq (three samples were excluded from the analysis because they had an alignment rate < 85%). RNA-seq data have been deposited in the ArrayExpress database at EMBL-EBI (www.ebi.ac.uk/arrayexpress) under accession number E-MTAB-7656. A summary of the RNA-seq data is provided in Additional file 1A. On average, ~ 90 million stranded 125-bp paired-end sequencing reads of each sample were aligned (Additional file 1A). Around 34,000 genes and 100,000 transcripts were detected per sample. Differential expression was analyzed with the DESeq2 and edgeR packages and genes were considered differentially expressed when both tests returned a significant result (cutoff: *P* < 0.01). RNA-seq data were validated by examining the expression patterns of established marker genes for sex determination and/or early sex differentiation in mice. Abundance of transcripts of many known important genes in the RNA-seq data was consistent with their documented expression patterns indicating the quality of our data. Examples of Sertoli cell genes included: *Gata4*, *Sry, Sox9, Sox10, Eno1, Amh, Fgf9, Dhh* and *Ptgds*; examples of granulosa cell genes included *Rspo1, Fst, Foxl2, Wnt4, Nr0b1, Irx3* and *Bmp2*; and examples of fetal Leydig cell genes included *Star*, *Hsd3b1, Cyp17a1, Cyp11a1* and *Prlr*. As a resource for the scientific community, we provide in a spreadsheet normalized expression levels from our expression analysis (see below) for all detected genes and transcripts (Additional file 2).

Two independent analyses were carried out. Firstly, differentially expressed genes (DEGs) between female and male gonads were identified (sex analysis at E11 and E12), and, secondly, DEGs were identified within each sex during the period of transition between sex determination and differentiation (time-course analysis between E11 and E12) (Fig. 1). In the sex analysis, 1228 and 1849 DEGs were identified in males vs females at E11 and E12, respectively (Table 1). At E11, corresponding to the onset of *Sry* expression in the XY genital ridge, 697 and 531 genes were upregulated in male and female gonads, respectively. The high number of genes expressed in a sexually dimorphic pattern at this early stage suggests that the sexual fate decision in the developing gonad depends on a complex network of interacting factors that converge at a critical threshold before *Sry* peak expression. At E12, 957 and 892 genes were upregulated in male and female genital ridges, respectively (Table 1). This increase in gonad gene expression at E12 corresponds to the differentiation and assembly of sex-specific cell lineages, and rapid sex gonad differentiation. Only 30 genes in males and 12 genes in females were commonly upregulated at E11 and E12 (Additional file 3E). In the time-course analysis, 3582 DEGs were identified in male genital ridges, of which 1897 were downregulated and 1685 were upregulated at E12 (Table 2). The fact that more genes were downregulated than upregulated suggests that transcriptional repression may play an important role at this stage of male gonad formation. Conversely, 7066 DEGs were identified in female gonads, of which 2882 were downregulated and 4184 were upregulated at E12 (Table 2). This increase in the number of DEGs in females has also been reported at E13.5 [12], indicating that a robust female-specific genetic programme is initiated at E12. Detailed information about detected DEGs and the complete spreadsheets containing the DEGs in every comparison can be found in Additional file 1B and Additional file 3, respectively.

**Table 1:** Summary of DEGs, DEIs and AS events detected (*P* < 0.01) in the sex analysis

		DEGs	DEIs	AS Events					
				3ss	5ss	ES	MIC	IR(U12)	Total
M vs F E11	Male Up	697	445	86	97	79	1	111(14)	374
	Female Up	531	619	93	125	180	3	843(70)	1244
	Total	1228	1064	179	222	259	4	954(84)	1618
M vs F E12	Male Up	957	977	123	130	185	2	218(29)	658
	Female Up	892	725	134	144	334	4	376(22)	992
	Total	1849	1702	257	274	519	6	594(51)	1650

*DEGs* differentially expressed genes, *DEIs* differentially expressed isoforms, *AS* alternative splicing, *3SS* alternative 3′ splice sites, *5SS* alternative 5′ splice sites, *ES* exon skipping, *MIC* micro-exon, *IR* retained introns

**Table 2:** Summary of DEGs and DEIs detected (*P* < 0.01) in the time-course analysis

		DEGs	DEIs
E11 vs E12 Male	E11 Up	1897	2282
	E12 Up	1685	2033
	Total	3582	4315
E11 vs E12 Female	E11 Up	2882	2548
	E12 Up	4184	3798
	Total	7066	6346

*DEGs* differentially expressed genes, DEIs differentially expressed isoforms

Genes that showed increased expression in E11 males were significantly enriched in RNA splicing, mRNA processing, translation and development Gene Ontology (GO) terms, indicating that splicing is important in male sex determination (Additional file 4A). In addition, functional annotation analysis indicated that cluster 4 encompasses processes found in the DAVID database of mRNA splicing and U12-type splicesomal complex (data not shown). Genes in E11 female gonads were enriched in cell migration and development GO terms (Additional file 4B). E12 male gonad genes were enriched in development, cell adhesion, male gonad development, and regulation of gene expression GO terms (Additional file 4C). E12 female gonad genes were enriched in development, lipid metabolic process, meiotic cell cycle and female gonad development GO terms (Additional file 4D). Many genes that were differentially expressed at E11 in males and females, were downregulated in their respective sex, and upregulated in the opposite sex at E12, indicating both the bipotentiality of many genes expressed in the gonads at E11 (Additional file 5) and the importance of specific downregulated genes in sex determination. The presence of autosomal genes showing sexually-dimorphic expression at E11 is consistent with the fact that sex differences exist in mammalian gonads before GSD has occurred [13, 14].

The sex and time-course analyses provided a baseline to identify genes showing sexually dimorphic expression at the E11 and E12 stages of gonadal sex determination and early differentiation. Our comparative analysis between sex and time-courses analyses of the genes upregulated in males or females at E11 and genes upregulated at E11 vs E12, indicated that 107 and 132 genes were specifically expressed in males and females, respectively, at E11 (Fig. 1C, E). In relation to the genes upregulated at E12 (just after peak *Sry* expression), we observed an increase in the number of genes solely expressed in female compared to male genital ridges (651 vs 490, respectively) (Fig. 1D, F). The spreadsheet containing the list of genes in every comparison can be found in Additional file 6. The majority of known male and female genes involved in sex determination are included in these lists, but the list also contains genes not previously related to GSD (Additional file 6). In addition, between E11 and E12, as expected for the arrival and division of germ cells, the upregulation of germ cell-specific genes was found (e.g. *Daxl*, *Nanog*, *Lefty1, Msx1, Ddx4, Sox2*).

To assess the early stages of sex differentiation, we compared genes that acquired sex-biased expression with those up- or downregulated at E12 (Additional file 7A). Nine-hundred and twenty-seven genes in males and 881 in females exhibited sex-biased expression at E12, respectively (genes with male- or female-biased expression in gonads at E12 but not at E11). For males, but not females, more genes were downregulated in the relevant sex than upregulated in the opposite sex, indicating that at this stage, transcription repression in males is important to establish male sexual differentiation. This trend was reversed in females; more genes were upregulated in females than downregulated in the males, suggesting a delay in the activation of the female gonadal development pathway.

Cluster analysis of DEGs at E11 and E12 was conducted using the Cluster Locator program [15] (http://clusterlocator.bnd.edu.uy). Clusters with 5 or more genes are represented in Additional file 8. Results indicated that at E11 (Fig. 1G) there was cluster enrichment in males, whereby 8 chromosomes (chrs) exhibited clusters containing more than 5 genes (between 5 and 10 genes), while in females there were 2 clusters of 6 genes (Additional file 8A, C). Of note, males showed the presence of a cluster with 10 upregulated genes. At E12, both males and females displayed significant enrichment of clusters (Fig. 1H). The number of clusters with more than 5 genes was higher in male than female gonads at E12, with males, as before, exhibiting a greater number of chromosomes with clusters (and genes within these clusters) than females (Additional file 8B, D). Also of note was the presence of 2 clusters of 6 genes and 1 of 5 genes on chr 10 in males at E12. Interestingly, most genes within a cluster followed a similar expression pattern. For example, in males at E12, expression of the genes in the first and third clusters on chr 1 (blue and red in Additional file 8 D) was increased at E12 versus E11, while those in chromosome 17 at E11 (pink in Additional file 8 C) were similarly expressed at E11 and E12. Another example is the two first clusters on chr 1 in males at E12, which exhibit an opposite pattern; in the first, expression was increased from E11 to E12 while the second there was a reduction in expression between these two time points (Additional file 8 D). Figures in Additional file 8 only display clusters containing at least 5 genes, but some smaller clusters may also prove to be interesting, such as 2 clusters of genes on chr 4 in females at E12 that appeared close together (*Rspo1, Sh3d21, Grik3*, and *Wnt4, Alpl*). The genes contained in these two clusters showed the same pattern of expression of *Rspo1* and *Wnt4* expression at E11 and E12 in both sexes. To analyse if theses clusters are conserved in other species, we analyzed if these genes are present in the previously described homologous synteny blocks (HSBs) on 10 amniotes (human, chimp, macaque, rat, mouse, pig, cattle, dog, opossum, and chicken) compared at < 1 human-Mbp resolution [16]. Between 44.7 to 61.3% of the genes that we found in our cluster analysis are also present in the conserved synteny blocks described by Larkin et al. (2009) (54.2 and 58.6% of the genes found in clusters in female at E11 and E12, respectively; and 44.7 and 61.3% of the genes found in clusters in male at E11 and E12, respectively) (Additional file 8 A-D). As a control, we generated 1000 random lists of the same size as the analyzed lists with genes selected randomly from the reference genome and we evaluated the percentage of genes included in HSBs. The percentage of genes included in HSBs was significantly higher in our lists of genes than in random gene lists of the same size in all the cases (*P* < 0.01). The results indicated that natural selection acts on the genome to maintain combinations of genes and their regulatory elements that are essential to sex determination and sex differentiation in mammals.

**Fig. 2:**
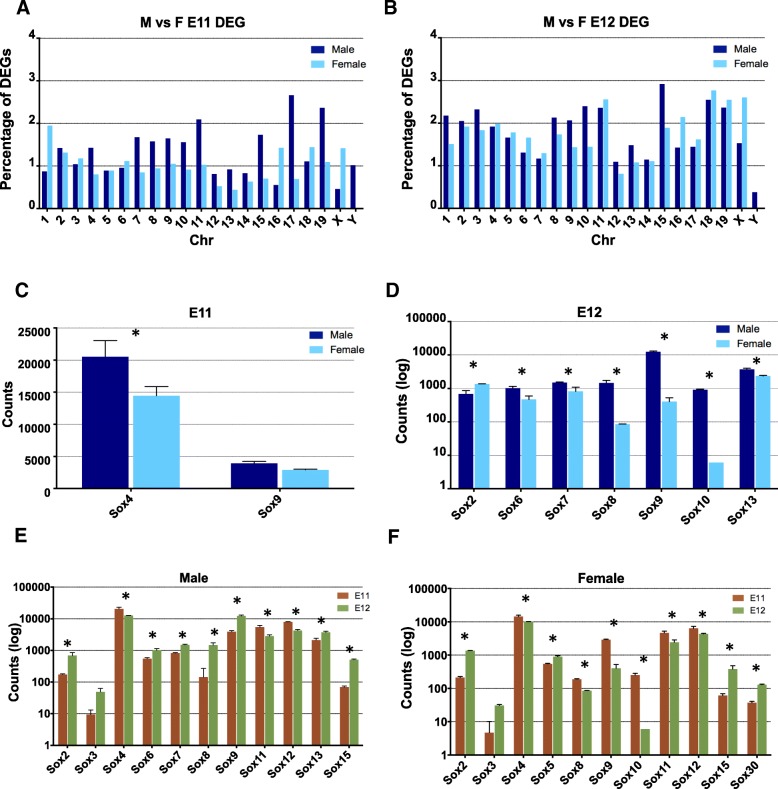
Chromosome distributions of differentially expressed genes (DEGs) and expression of *Sox* family genes. Relative chromosome distributions in percentages of DEGs in E11 (**a**) and E12 (**b**) male and female gonads. Differential expression in counts of Sox family genes in the sex analysis (**c**, **d**) and in the time-course analysis (**d**, **f**). Error bars represent the standard deviation. Bars with different superscripts differ significantly (*p* < 0.01)

The distribution of the mapped genes according to the total number of genes on each chromosome are indicated in Fig. 2 A,B. Chromosomal distributions of 1228 (697 in males and 531 in females) DEGs between males and females at E11 (Fig. 2A) and 1849 (957 in males and 892 in females) at E12 (Fig. 2B) revealed enrichment of DEG mappings at E11 for males to chrs 11, 17, 19 and Y, and for females to chrs 1, 16, 18 and X and at E12 for males to chrs 1, 9, 10, 15 and Y, and for females to chrs 16 and X. These differences between clusters and enriched chromosomes of genes reveals differences between genes related to sex determination at E11, and differences between genes related to sex differentiation at E12. Further, the presence of gene clusters points to the idea that genes related to sex determination and sex differentiation are synergistically orchestrated in gene neighbourhoods with particular characteristics including the transcriptional co-regulation of multiple genes.

Among the families of genes detected, the Sox family of genes was particularly interesting. In E11 gonads, sexually dimorphic expression of *Sox4* was observed, a regulator of gonad morphogenesis in mice [17], while *Sox9* showed similar expression (Fig. 2C). In E12 gonads, *Sox2* expression was greater in females and the expression of *Sox6*, *7*, *8*, *9, 10* and *13* was greater in males (Fig. 2D). When comparing stages E11 and E12, 14 genes of the Sox family were also differentially expressed: *Sox8* and *9* expression levels increased between the two stages in males and decreased in females. *Sox10* expression decreased in females. Increased *Sox6*, *7* and *13* expression was only observed in males while increased *Sox5* and *Sox30* expression was observed in females (Fig. 2 E, F). Moreover, of the 2612 genes analyzed on the X chr at E11, only 37 genes were upregulated in females. In addition, 12 genes on the X-chr and 16 on the Y-chr (out of 1570 analyzed on the Y chr) were also upregulated at this time-point in males (Additional files 2 and 3). At E12, 68 genes on the X-chr were upregulated in females, and 40 genes on the X-chr and 7 on the Y-chr were also upregulated in males (Additional files 2 and 3).

### Gene isoform expression during sex determination in mice

**Fig. 3:**
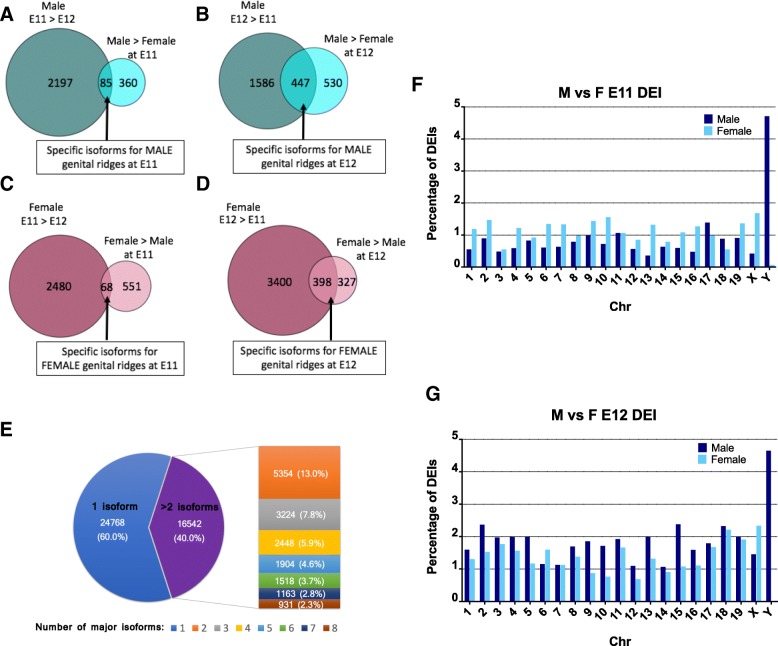
Differentially expressed isoforms (DEIs) in E11 and E12 male and female gonads and their chromosome distributions. (**a-d**) Venn diagrams showing DEIs in E11 and E12 male and female gonads. Gene subsets represent genes specifically upregulated in E11 (**a**) and E12 (**b**) males, and E11 (**c**) and E12 (**d**) females. (e) Number (and proportion % from total) of genes with different number of distinct major isoforms. **(f, g**) Relative chromosome distributions in percentages of DEGs in E11 (**f**) and E12 (**g**) male and female gonads

Differentially expressed isoforms (DEIs) were analyzed using edgeR applied to transcript-level estimated counts obtained from the Salmon software [18]. The number of isoforms detected (cutoff: *P* < 0.01) are shown in Additional file 1C. As for the previous DEG analysis, two independent analyses were carried out, one to identify DEIs between female and male gonads (sex analysis at E11 and E12) (Fig. 3 A-D), and the other to identify DEIs within each sex during the period of sex determination and differentiation (time-course analysis between E11 and E12) (Fig. 3 A-D). At E11, 1064 DEI were identified, 445 upregulated in males and 619 in females (Table 1). At E12, 1702 DEI were found, 997 upregulated in males and 725 in females (Table 1). The time course analysis identified 10,661 DEIs between E11 and E12, 6346 DEIs in females, and 4315 DEIs in males (Table 2), indicating that in females there is a greater difference between the isoforms expressed in the transition between E11 and E12 than in males. Only 63 isoforms in males and 9 in females were commonly upregulated at E11 and E12 (Additional file 9E). We found 110,330 transcripts representing 41,311 genes, and over 40% of these genes (16,542) expressed multiple isoforms (Fig. 3E), indicating extensive alternative splicing in mouse developmental gonads. Detailed information about detected DEIs and the complete spreadsheets containing the DEIs in every comparison can be found in Additional file 1C and Additional file 9, respectively.

**Fig. 4:**
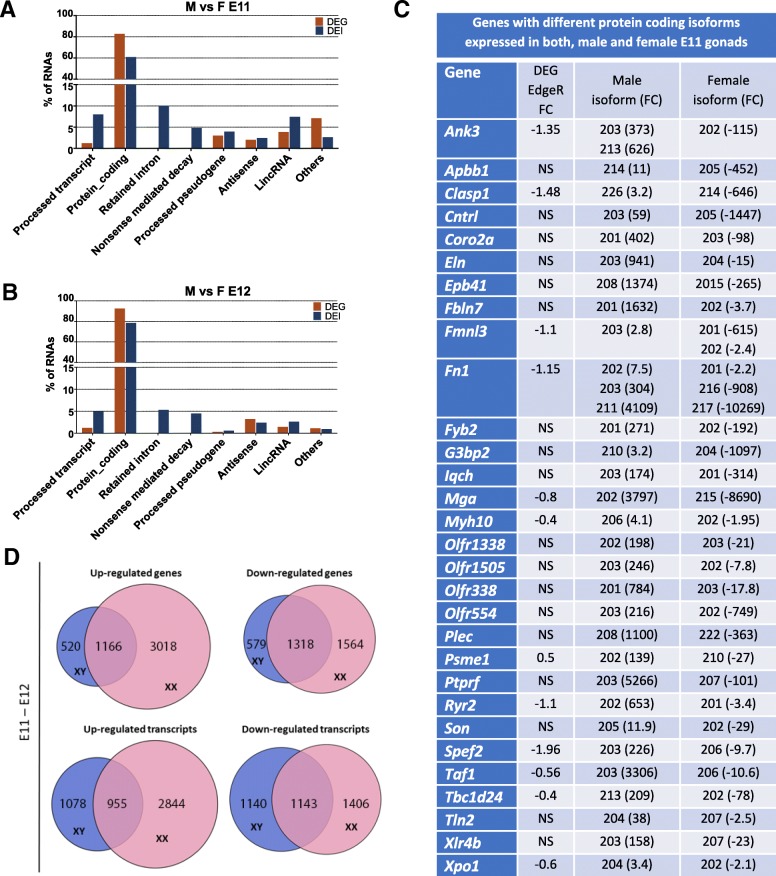
Differences between RNA types and numbers of genes identified in the differentially expressed gene (DEG) and differentially expressed isoform (DEI) analyses and list of DEIs detected in E11 males versus females. Differences in RNA revealed by the DEG versus DEI analysis in E11 (**a**) and E12 (**b**) gonads. (**c**) Genes with different isoforms expressed in both male and female E11 gonads. (**d**) Venn diagrams comparing up- or down regulated genes in XX and XY gonads during the E11 to E12 interval

Comparison between DEGs and DEIs revealed that at E12, ~ 69% of genes and isoforms were common; in addition, a high level of common events was detected in the time-course analysis between E11 and E12 for females (~ 78%) and males (~ 70%). However, a small proportion of DEIs identified at E11 were recognized in the DEG analysis (~ 19%) (Additional file 1D and Additional file 10). This indicates the presence of multiple isoforms at E11 in genital ridges that are different to the sum of the expression of all the isoforms of a gene, and in some case, different to the principal isoform expressed. This is in contrast to what occurs at E12 and in the time-course analysis between E11-E12, where common events are greater because the DEIs coincide with the higher expressed isoform and/or with the sum of all the isoforms of that specific gene. Differences between gene and isoform analysis results could also be related to the type of transcript identified (Fig. 4 A,B) because DEG analysis does not differentiate between protein coding and retained intron or nonsense-mediated decay. Hence, the amount of protein coding identified by DEG analysis was higher than that identified by DEI analysis. In addition, the number of processed transcripts not containing an ORF or LincRNA (long interspersed ncRNA, non-coding RNA) was greater in the DEI analysis.

To examine the relative contributions of transcriptional activation and repression to the establishment of sex-determining DEI, we compared genes that exhibited sex-biased expression with those regulated transiently during the interval from E11 to E12 (Additional file 7B). Results were similar to those obtained in the DEG analysis. Only in males were more genes downregulated than were up regulated in the opposite sex, indicating that at this stage transcriptional repression is important for establishing male sexual differentiation.

Our ontology analysis yielded similar results to those obtained for genes in the case of males but not females (Additional file 11). Genes showing increased expression at E11 in males were significantly enriched in transcription regulation, translation and development Gene Ontology (GO) terms (Additional file 11A). E11 female gonad genes were mainly enriched in G-protein coupled receptor signalling pathway, sensory perception, and cell adhesion GO terms (Additional file 11B). In both male and female gonads at E11, functional annotation analysis indicated clusters that encompassed processes found in the DAVID database of mRNA splicing (data not shown). E12 male gonads were mainly enriched in development, negative regulation of canonical Wnt signalling pathway, and male gonad development GO terms (Additional file 11C). E12 female gonads were mainly enriched in development, cell differentiation, Wnt signalling pathway, and negative regulation of transcription from RNA polymerase II promoter GO terms (Additional file 11D).

Comparative analysis between isoforms upregulated in males vs females at E11, and isoforms upregulated at E11 vs E12, indicated that 85 and 68 subset of isoforms were uniquely expressed in males and females at E11, respectively (Fig. 3A,C and Additional file 12), 38 and 19%, respectively, of which corresponded to isoform 1. Within the subset of isoforms upregulated at E12, 447 and 398 were specifically expressed in males and females, respectively (Fig. 3B,D and Additional file 12); 52 and 57%, respectively, of which corresponded to isoform 1. In contrast to DEG, at E11, just before the *Sry* expression peak, we observed an increase in the number of unique isoforms preferentially expressed in female compared to male genital ridges (619 vs 445, respectively). We then went on to examine the temporal dynamics of gene and isoform expression by comparing DEG and DEI between E11 and E12 (Fig. 4D and Additional file 13). During this transition between sex determination and sex differentiation, more genes and isoforms in male gonads were downregulated than upregulated, suggesting that transcription repression may play an important role in early-stage male gonad differentiation. However, in female gonads, more genes and isoforms were upregulated than downregulated, suggesting that an intense ovary-specific transcription programme takes place right from the onset of fetal gonad development.

Interestingly, 30 genes at E11 and 22 at E12 showed different DEI in males and females (Fig. 4C), some of which are related to important functions in sex determination in E11 gonads: control of mRNA splicing (*Son*: Son DNA binding protein), control nuclear-cytoplasmic transport (*Xpo1*: exportin 1), repressor of germ cell-related gene expression (*Mga*), and testis-specific splicing (*Taf1*: TATA-box binding protein associated factor 1, on chr X). The male isoform of *Son* loses the Son domain in the protein, a region of multiple unique repetitive sequence motifs of unknown function that span nearly 1200 amino acids. The female isoform of *Xpo1* loses the UTR region (containing Sry, Sox and Musashi binding element); the female isoform of *Mga* loses the last 2 exons (~ 500 amino acids) that contain the helix-loop-helix DNA-binding domain; the first intron of the male isoform is 4 kb shorter than the corresponding female isoform; and the male isoform of *Taf1* retains an intron. Examples of DEI in the E11 gonad previously not related to sex determination that could have a role in this process are: exosome complex component mtr3 (*Exosc6*) involved in RNA processing and degradation, and karyopherin Subunit Alpha 2 (*Kpna2*) involved in gonad development [19]. Examples of DEI in E11 gonads previously not associated with sex determination that could play a role in female sex determination are: mediator complex subunit 1 (*Med1*) co-activators to direct transcriptional initiation by the RNA polymerase II apparatus; transforming growth factor, and beta receptor I (*Tgfbr1*) whose over-activation drives gonadal tumor development [20].

### Alternative splicing events during sex determination in mice

Alternative splicing events were analyzed using vast-tools software to categorize events into 5 different groups: 3′ AS site (3SS), 5′ AS site (5SS), exon skipping (ES), intron retention (IR) and micro-exon (MIC) based on their inclusion levels. Overall, 1648 differentially spliced events were identified between males and females at E11, 1244 upregulated in females and 374 in males. At E12, 1650 events were differentially spliced between males and females, 992 upregulated in females and 658 in males (Table 1 and Additional files 14, 15, 16 and 17). At both E11 and E12, a greater number of AS events were upregulated in females. The events principally accumulated in females were IR and ES. In E11 female gonads, we found 843 IR events compared with 111 in E11 males.

**Fig. 5:**
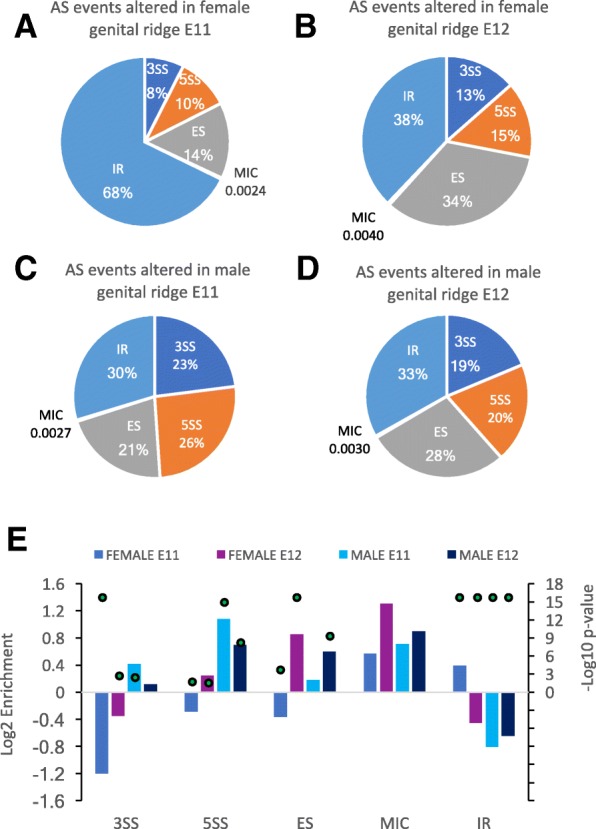
RNA-seq analysis of alternative splicing in male and female gonads. Categories of alternative splicing (AS) events upregulated in female E11 (**a**) and E12 (**b**) gonads and male E11 (**c**) and E12 (**d**) gonads. (**e**) Log2 values of fold enrichment (normalized to the overall distribution of event categories in mouse transcriptomes) and –log10 of fold enrichment *p* values are provided for each AS category in the lower panel. 3SS, alternative 3′ splice sites; A5SS, alternative 5′ splice sites; ES, exon skipping; MIC, alternative micro cassette exon ≤15 nucleotides; IR, retained introns

**Fig. 6:**
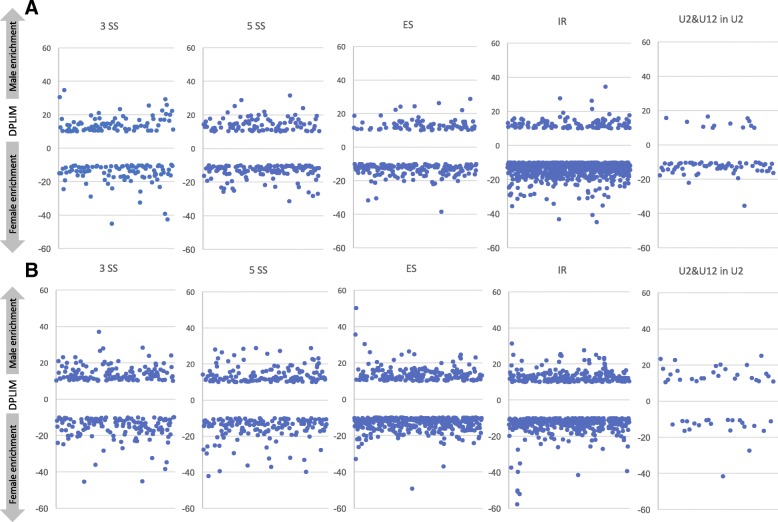
Alternative splicing (AS) events in male and female E11 and E12 gonads. Number and fold changes of AS events in the gonads of males compared to females at E11 (**a**) and E12 (**b**) (measured as log2 ratio of DPLIM read counts in males versus females). AS category: 3SS, alternative 3′ splice sites; A5SS, alternative 5′ splice sites; ES, exon skipping; IR, retained introns; U2&U12inU2, U2 IR plus U2 intron in U12-containing genes

Distributions of AS events differed between male and female genes. In E11 female genital ridges, a higher significant proportion of IR events (68%) was observed compared to E11 male samples (30%) (Fig. 5A,C; Additional files 14 and 16). Conversely, a higher proportion of 3SS (23%), 5SS (26%) and ES (21%) events were observed in E11 male samples. At E12, the most frequent AS event in female samples was IR (38%) followed by ES (34%), 3SS (13%) and 5SS (15%). In E12 male samples, AS event distribution was similar to that observed at E11 (Fig. 5B,D; Additional files 15 and 17). Once normalized according to the overall distribution of AS events in the mouse, 3SS events were under-represented in female samples compared to male samples (both at E11 and E12) (Figs. 5E and Fig. 6). E11 and E12 male samples showed greater enrichment in 5SS events compared to female E11 and E12 gonad genes. IR events were found to be under-represented in male samples compared to female samples at E11, where slight enrichment was observed (Fig. 6A). At E12, ES events were also over-represented in male and female gonads (Fig. 5E). We also observed an enrichment in U12 type intron retention (genes with U12 type intron) in both sexes, showing greater enrichment in females at E11. In addition, comparisons were made between male and female AS events to find common genes showing different splicing events between sexes. Ninety-two genes with 228 different splicing events were identified in both males and females at E11, and 149 genes with 358 different AS events were identified in both males and females at E12. Of 3755 sex-biased AS events observed, only 277 were common to both time points E11 and E12 (Additional file 18).

Among the genes showing different AS events in male and female gonads at E11 and E12, functional annotation analysis (DAVID analysis) indicated the presence of 41 genes related to transcription regulation, 27 related to zinc-finger, 15 related to Ubl conjugation pathway, 11 to RNA binding, 3 with mRNA splicing, 14 with protein transport, 9 to spermatogenesis, 10 to cellular response to DNA damage, and 6 to histone deacetylase (Additional file 18 D). It is important to highlight that some of these genes, for example *Chtf18*, have been associated with germ cell proliferation in the fly and mouse, and with male infertility [21]; *Huwe1*, interacts with *Gadd45g* (essential for primary sex determination) and regulates the establishment and maintenance of spermatogonia [22, 23]; the splicing factor *Setx* has been related to infertility in males and reduces fertility in females [24]; Igf2 has potential physiological functions in male and female gonads [25]; *Tspan33* is a candidate anti-testis gene expressed independently of both *Foxl2* and *Wnt4* [26]; *Dock9* (guanine nucleotide exchange) has been identified as enriched regulatory motifs in the promoters of putative Sox8/Sox9-dependent genes [27]; and *Chtf18* (chromosome transmission fidelity factor 18) causes impaired spermatogenesis in mice [21].

## Discussion

Sex determination is achieved through a vast array of mechanisms and it is assumed that transcription level regulation drives this cascade of mechanisms in mammals. However, transcription factors can alter gene expression beyond transcription initiation by controlling pre-mRNA splicing and thereby mRNA isoform production [8]. While it seems that post-transcriptional mRNA splicing regulation mechanisms are a common theme among distant phyla, from insects, such as flies, honeybees, or wasps (6), to reptiles (7), their role in mammals is still poorly understood. Our study provides new information about novel isoforms and alternative splicing events that appear to be essential for gonadal sex determination and early sex differentiation. Our data also identify new candidate genes that could be involved in disorders of sexual development.

While most murine multi-exon genes can be spliced into multiple transcript isoforms, the functions of the majority of AS events are not known [28]. In effect, for each of the 25,000 known protein-coding genes, there are at least 10 more isoforms [29], and it seems that at least 48% of multi-exon protein-coding genes express multiple splice variants that are highly regulated in a cell/tissue-specific manner [30]. However, the number of reported transcripts does not fully represent all the alternative splicing events in a tissue. The Ensembl database use annotates transcripts based on experimental evidences. Thus, to examine the role of alternative splicing in sex determination and/or early sex differentiation in the mouse, we first analyzed DEIs related to Ensembl-annotated transcripts using an alignment–independent method (*Salmon* software). We then determined the presence of other non-annotated AS isoforms using a RNA-seq analysis pipeline developed using an alignment-dependent method (*vast-tools*) [30]. This software package was designed to identify various types of AS events such as alternative exon skipping, alternative usage of splice sites and intron retentions.

Several RNA-seq analysis studies have examined gene expression in gonads at E10.5, E11.5 and E12.5 [31–33]. Our study is the first to focus on two time points (E11 and E12) close to the peak of *Sry* expression (at E11.5) in an effort to obtain novel information on sex determination and sex differentiation. Despite differences in experimental design, a high proportion of the sexually dimorphic genes reported by Zhao et al. [32] at E11.5 and E12.5 were detected in our DEG study at E12 (Additional files 19 and 20) which validates the study. However, in addition we were also able to detect hundreds of DEGs in the gonads of E11 males and females at that were not present in the analysis of Zhao et al. [32] at E10.5 or E11.5 (Additional files 19 and 20). Moreover, we also identified many novel isoforms at both E11 and E12 (Additional files 21 and 22), likely due to our higher read count (we detected ~ 100,000 transcripts vs ~ 19,000 by Zhao et al. [32]). In addition, this is the first study to explore alternative splicing during sex determination and differentiation in mice using *vast-tools* to comprehensively detect and quantify all major types of AS events from raw RNA-seq reads. Both analyses are required to identify post-transcriptional splicing events, as only 10% of the genes identified in the differential isoform and differential AS analyses were common to both tests (Additional file 23). Deep RNA-seq analysis allowed us to precisely determine gene-, isoform- and AS level expression patterns at the time of sex determination and early sex differentiation in the murine gonads.

Our DEG data revealed different early sex markers present in male and female E11 gonads. At this embryonic stage, XY gonads maintain the ability to initiate *Sox9* activation upon *Sry* expression [11], while XX gonads maintain the ability to differentiate into female ovaries in the absence of *Sry* expression. We discovered thousands of DEGs in males and females at E11 vs E12, indicating a switch between bi-potential and sex differentiating gonads, and found hundreds of DEGs in male vs female gonads at E11 (1228 genes) and even later at E12 (1849 genes). Considering the specific genes that were more highly expressed in males or females at E11 and that were subsequently downregulated at E12, we identified 107 upregulated genes specific to males and 132 specific to females at E11, indicating that, at this early stage, a greater number of genes specific to female sex determination are expressed. Also, at early differentiation, considering the specific genes that were more expressed in males or females at E12, we identified 490 genes upregulated at this time point specific to males and 651 specific to females. These genes represent good marker candidates of early events in sex determination that are potentially involved in disorders of sexual development (DSD). In effect, 13 of the 15 novel candidate genes that were recently reported to play a role in 46XY DSD [34] were present in the list of 490 genes specific to E12 males, and the other 2 genes were found to be more highly expressed at E12 than E11 (Additional file 6). Many of these genes showing sexually-dimorphic expression at E11 are autosomal, pointing to sex differences before gonadal sex determination induced by *Sry*. For example, high expression of *Sox4* is observed in E11 males before the upregulation of *Sox9*. It was recently reported that *Sox4* regulates gonad morphogenesis and promotes male germ cell differentiation in mice [17] suggesting repressed transcription of the sex-determining gene *Sox9* via an upstream testis-specific enhancer core element (TESCO) in fetal gonads. Interestingly, some members of the Sox family, such as *Sox5, Sox6* and *Sox13*, which possess a leucine zipper and a coiled-coil domain causing them to form constitutive dimers in solution, interacting with a spliceosomal complex, have been recently implicated in mouse testis development and in sex determination in the teleost medaka [35, 36]. Moreover, this list of genes includes numerous genes for which expression has not previously been described as being sexually dimorphic in mammals, including 29 genes related to mRNA splicing (Additional file 4A) and 11 genes related to U12-type spliceosomal complex (*Lsm7, Phf5a, Snrnp25, Snrpb, Snrpd1, Snrpd2, Snrpd3, Snrpf, Sf3b6, Sf3b5, and Zmat5*). This complex only processes 0.5% of human or murine introns but is conserved in metazoan organisms and shows evolutionary conserved positions within its host genes, not only within vertebrates, but in some cases also in invertebrates [37], with functions in development [38] and a possible role in early sex-developmental pathways in mammals [39].

In addition, some discrepancy was found here in the expression of certain genes, such as *Fgfr2*, which has been described as the receptor for *Fgf9* in the XY gonad and ensures sustained *Sox9* expression through repression of ovarian pathways [40]. However, we observed higher expression levels of *Fgfr2* in E12 females than in males. Interestingly, only 4 isoforms of the 17 coding isoforms of *Fgfr2* displayed high expression in females, indicating that, in addition to the role that some isoforms can have in male GSD [40], others may play a critical role in female GSD, and that the current classification of *Fgfr2* in 2 isoforms may need to be updated. We also observed greater expression of *Wt1* and *Amhr2* in E12 female gonads, probably related to the contribution of *Wt1* to female sex differentiation (in addition to its role in gonad development and male sex determination). We identified 8 of the 9 isoforms of *Wt1*, 4 with +KTS and 2 –KTS. We also found E12 female upregulation of 2 *Wt1os* (Wilms tumor 1 homolog, opposite strand) antisense isoforms (*Wt1os-201* and *− 202*)*,* suggesting a possible role in post-transcriptional control of *Wt1* expression*.* In addition, there is evidence to suggest that *Wt1* regulates alternative splicing [41] and that *Wt1* knockout mice exhibit reduced expression of many splicing factors including *Zrsr1, U2af1, Son, Srsf5, Sf3a1* and *Rsrc1* [42].

The early molecular mechanisms determining female gonad sex determination and early sex differences in the male gonad before *Sry* expression at E11 are now relatively well understood. Some differences may be due to sex chromosome contributions. Our data indicate that at E11 and/or E12, when expression of *Sry* is low, several genes localized within a 1 Mb region of the Y chromosome are highly expressed (*Eif2s3y*, *Uty*, *Kdm5d*, *Uba1y*, *Ddx3y, Usp9y, Zfy1*). Sexually dimorphic expression of these genes has been reported [12]. Some have a homologue on the X chromosome, and the sum of the expression of the two genes leads to the greater expression in males of *Ddx3y/x* than the two *Ddx3x* in females. The same occurs with *Eif2s3y/x* while the opposite occurs for the histone demethylases *Uty/x* and *Kdm5d/c*, and there are no differences for *Uba1/y*. Moreover, several genes on the X chromosome are more highly expressed in females at E11 than in males (*Xist*, *Tsix*, *Pgr15l*, *Rlim*, *Kdm5c*, *Kdm6a*, *Usp9x*, *Pola1*, *Hcfc1*, *Elf4*, *Eif2s3x*, *Tab3*, *Shroom4*, *Amot*, *Taf1*, *Fmr1*, *Flna*, *Clcn5*, *Usp26*, *Tro*, *Pet2*). Some have a homologue on the Y chromosome, but if we sum the expression of both genes of the Y and X chromosomes, females exhibit higher expression than males (with the exception of *Eif2s3y/x* which is more highly expressed in males). The majority of these genes are related to ubiquitination (*Rlim*, *Kdm6a*, *Usp9x*, *Clcn5*, *Usp26*), transcription factors and regulators (*Pola1*, *Hcfc1*, *Elf4*, *Eif2s3x*, *Tab3*, *Taf1*, *Pet2*), and chromosome modifications (*Xist*, *Tsix*. *Kdm5c*, *Kdm6a*, *Fmr1*). Interestingly, some X chromosome genes are more expressed in males than females (*Emd*, *Cox7b*, *Rpl39*, *Bex1*, *Xiap*) (Additional file 3). Surprisingly, at day E12, 62 genes escaped X-inactivation and were upregulated in females, while 38 X-chromosome genes were upregulated in males. This is consistent with previous data indicating that coding sequences of X-linked genes showing germline expression are disproportionately shaped by positive selection [43]. In *Drosophila* and *C. elegans*, the process of sex determination is directly linked to X-chromosome dosage compensation. In contrast, in mammals, it is not known how dosage compensation is linked to sex determination. However, it has been recently reported that in mouse and human germlines, X dosage compensation states are determined by the X chromosome, not by phenotypic sex [44]. In addition, sexually dimorphic expression of chromatin modifiers could affect the activity of many genes leading to sex-specific transcriptional programmes allowing XX and XY bipotential gonads to initiate differentiation with different chromatin landscapes [45].

Regarding the cluster analysis, the results suggest that genes involved in sex determination and early differentiation pathways could be synergistically regulated in specific clusters with characteristic features. One comparative genomic study of amniotes by Larkin et al. (2009) [16] found that some chromosomal regions have been reused during the mammalian chromosomal evolution and regions with few breakpoints tended to include genes for development. Around 50% of the genes that we have found in our cluster analysis are also present in the conserved syntenic blocks described by Larkin et al. (2009) indicating a selective advantage and an evolutionary conservation of these clusters related with sex determination and sex differentiation even in distant mammalian and amniote species.

Our results reinforce the idea that potentially important isoform-level genetic changes can be missed by gene-level measurements. In this study, only 20% of events were common to DEG and DEI analyses in male and female E11 gonads (but not at E12 or during the transition between E11 and E12) suggesting the importance of specific isoforms in sex determination (Additional file 1D and Additional file 24). DEIs in E11 males not identified in the list of DEGs included 49 isoforms of transcription regulators, 6 of intracellular protein transport, 6 of positive regulation of the canonical Wnt signalling pathway, and 4 of mRNA splicing. DEIs in E11 females not identified is the list of DEGs included 12 isoforms of protein transport, 30 of transcription regulators, 12 of protein ubiquitination and 3 of mRNA splicing. We identified several isoforms differentially expressed in male and female E11 gonads that may be important for sex determination in mice, including *Mga*, *Son* and *Xpo1*. The transcription factor *Mga* (MAX Dimerization Protein), which forms a complex with *Max* and represses germ cell-related gene expression in mice [46], was differentially expressed in males and females at E11. The isoform *Mga.202* was expressed in E11 males, while in females, the *Mga.215* isoform was expressed, which differs from the former in that it lacks the last two exons coding for a helix-loop-helix DNA-binding domain. Differences in *Son* isoforms at E11 could be important for sex determination. *Son* is highly expressed and is an essential factor in embryonic stem cells, required for proper RNA splicing of selective genes [47]. The functional significance of a short *Son* isoform has been recently reported [48]. Increases in short splice variants of *Son* in acute myeloid leukemia antagonize full length *Son* function in transcriptional repression of leukemia-associated genes [48]. We also identified differences in isoforms of *Xpo1* in males and females at E11. These differences can lead to a differential balance between nuclear import and export in gonads and drive the developmental programme towards a male or female. A high level of nucleocytoplasmic trafficking has been reported in the gonad consistent with sex determination and the exportin subtype *Xpo1* showed the highest signal values during fetal testis development between E11 and E18 [49]. In humans, there is total dependence of male sex determination on nuclear transport of SRY and SOX9. Mutations in SRY cause defective nuclear localization signal recognition by importins, failure in SRY nuclear accumulation, and XY sex reversal [50]. In addition, defects in SOX9 nuclear import can cause sex reversal [51]. Mouse gonad exposure to the Xpo1-mediated nuclear export inhibitor leptomycin B (LMB) around the time of sex determination gives rise to SOX9 in the nucleus of germ cells leading to the formation of testis cord-like structures, providing evidence that export of Sox9 is critical for female gonad determination [52]. The potential functions of DEI such as *Mga*, *Son* and *Xpo1* isoforms in sex determination and gonad development warrants further investigation.

The important role of AS in sex determination in mice is indicated by significant enrichment in RNA splicing of genes showing increased expression in E11 males (*Zmat5, Magoh, U2af2, Snrpd3, Cwc15, Gm3376, Snrpd1, Snrpd2, Snw1, Sf3b6, Sf3b5, Smndc1, Eif4a3, Ppih, C1qbp, Ppp1r8, Frg1, Prpf8, Snrpb, Snrpa, Lsm4, Lsm3, Phf5a, Snrpf, Snrnp25*), and of the subset of DEG upregulated in E12 females (*Wt1*, *Fmr1*, *Mbnl3*, *Srsf12* and *Zrsr2*). This important role is also reflected in the differences in the amounts and types of splicing events observed between males and females at both E11 and E12. Our results indicate that intron retention (IR) is the principal AS event upregulated in E11 females and downregulated in E11 males, and in E12 stage males and females (Table 1, Figs. 5 and 6). The IR mechanism modulates gene expression levels in multiple biological processes during normal development as well as in response to stress and disease [53]. IR occurs when splicing machinery fails to excise introns and may lead to the inclusion of premature termination codons, decreasing the expression of genes that are less required or not required for the physiology of the cell or tissue type, but the exact function of IR remains poorly understood. Ontology analysis of the 250 IR events that were more upregulated in E11 females indicated enrichment for the biological processes of regulation of transcription (44), cell cycle (15), protein phosphorylation (14), protein transport (5) and mRNA splicing (*Cd2bp2, Khdrbs2, Rbm5, U2af1l4, Zfp326*), and enrichment for the molecular process of nucleic acid binding (31) and protein kinase activity (15). Recently, it has been reported that the Y chromosome modulates splicing and sex-biased IR rates in *Drosophila* [54]. Here, we observed that male and female gonads before the *Sry* peak (at E11) show a large difference in IR, highlighting the significance of sex-biased IR in tuning sex differences. In *Drosophila*, sex lethal (SXL) protein binds to the male specific lethal-2 (*MSL2*) 5’UTR inhibiting the splicing of a resident intron regulating sex determination [53]. IR of *JARID2* (a *Jumonji* family gene) is implicated in reptile temperature-dependent sex determination and sex reversal [7]. Here, we found AS enrichment of *Jarid2* in E11 males. In mammals, a closely related *Jumonji* family member (*Kdm3a*) is a direct regulator of SRY, and *Kdm3a* dysfunction causes male-to-female sex reversal in mice [55]. We observed upregulation of *kdm3a*-203 in E11 females and of *kdm3a*-201 in E12 females, suggesting a possible role of *kdm3a* in female sex determination.

Female E11 gonads also featured U12 IR enrichment affecting both U12 introns and U2 introns of genes with some U12 introns (Additional file 1E and Additional file 16). U12 or minor type-introns are a minor subgroup of introns, distinct from the major or U2-type introns. U12 type introns are a rare class of introns present in most eukaryotes (only 0.5% of all introns) that are processed by a specific U12-dependent spliceosome, and are mostly found arising from the same orthologous genes despite large evolutionary distances [56]. They are important in development, disease, and cell division [39]. There is evidence to suggest that U12-type introns are essential for the regulation of gene expression [8, 37]. The presence of U12 in high levels in E11 females suggests a role for post-transcriptional gene regulation in this early stage of female sex determination.

The second AS event upregulated in female gonads was exon skipping (ES), an important mechanism through which cells expand their transcriptome and proteome repertoires, creating different splice variants with distinct cellular functions used by a wide range of essential genes [3]. In addition, transcripts showing abnormal exon skipping are actively degraded [57], and abnormal transcription (faster or slower than normal) contributes to increased ES [58]. Our results highlight the events of ES (of 6 different exons) of histone-binding protein *Rbbp4* in E11 females. *Rbbp4* is a component of polycomb repressive complex 2 (PRC2) with similar DEG in males versus females at E11 and E12, but higher numbers of DEI in E11 females. The higher proportion of ES in *Rbbp4* in females suggests that the gene is not active at this stage and that PRC2 repression could be reduced, allowing for the expression of genes potentially related to female sex determination, similar to the effect of *Cbx2* in males*,* as an activator of *Sry,* by targeting polycomb repressive complex 1 (PRC1) [59]. Interestingly, other components of PRC1 were upregulated in E12 females (*Scmh1* and *Pcgf5*) and high expression of *Kdm3a* (required for activation of *Sry* in males at E11) was observed in E12 females, suggesting a role in female sex determination. Consistent with this idea, if we consider the 60 genes showing the highest ES value, 5 genes related to the chromatin repressor exhibited higher incidence of ES in E11 females: BCL6 co-repressor-like 1 (*Bcorl1*), breast cancer 1, early onset (*Brca1*), histone deacetylase 9 (*Hdac9*), retinoblastoma binding protein 4 (*Rbbp4*) and AT rich interactive domain 5A (MRF1-like) (*Arid5a*). In addition, 3 genes related to KRAB-containing zinc-finger repressor proteins showed upregulated ES in E11 females: expressed sequence AI987944 (*AI987944*), zinc finger protein 58 (*Zfp58*) and zinc finger protein 81 (*Zfp81*). However, none of these genes showed ES upregulation at E12 (considering the 120 genes with the higher ES values). Also, ontology analysis of these ES upregulated at E12 only identified enrichment of genes related to protein binding, and 7 genes related to transcription regulation. These results suggest that upregulation of IR and ES events in genes related to chromatin repression in E11 females is a post-transcriptional mechanism that may control sex determination in females. Using this mechanism, bivalent pre-granulosa and pre-Sertoli cells can show similar gene expression and differences in AS may act in combination in the divergence of sex determining pathways, maintaining genes in a poised state and using AS for rapid activation or repression upon differentiation. Besides the production of specific isoforms of proteins involved in sex determination/differentiation, intron retention could be a powerful mechanism to finely regulate the stoichiometry of active proteins without modifying transcription.

## Conclusions

In conclusion, our data suggest a need to carefully analyze isoform variants and AS to understand complex processes such as sex determination and differentiation. This study highlights significant differences in mRNA splicing between males and females, suggesting that important steps in the process of mammalian sex determination are likely to operate at the post-transcriptional level, and that splicing regulatory mechanisms are a common feature of sex determination in distant phyla. Our study contributes new information concerning the transcriptional landscape and mRNA splicing in sex determination and early sex differentiation and provides a list of isoforms that should be functionally validated to confirm their role in these processes and in processes related to mammalian disorders of sexual development.

## Methods

### Embryo generation and tissue collection

Animal experiments were performed in accordance with the recommendations of European Community Council Directive 2010/63/EU guidelines. Experiments were approved by the Committee on the Ethics of Animal Experiments of INIA (permit number CEEA 2012/021). To generate embryos, we used CD-1 mice (Harlan Iberica SL). Mice were provided a standard diet ad libitum and kept in a temperature- and light-controlled environment (22–24 °C, 14 L:10D). After mating, mice were euthanized by cervical dislocation and fetuses were collected on embryonic days 11 (E11) and 12 (E12). At each time-point, individual pairs of gonads were collected. The sex of the fetuses was determined by PCR on tail DNA using primers for *Smcx/Smcy* [60] as, at E12, males and females are morphological indistinguishable. Genital ridges were dissected away from the mesonephros in sterile PBS and stored at − 80 °C until all samples were collected. Three pairs of gonads from different animals were grouped to produce three samples (biological replicates) of each sex at E11 and E12 for RNA-seq analyses, and three sets of samples for qPCR analysis.

### RNA extraction

RNA was extracted using a Direct-zol™ RNA MiniPrep Kit (Zymo Research, CA, USA) following the manufacturer’s protocol. DNA digestion was performed with RQ1 RNase-Free DNase (Promega, WI, USA) [61]. Total RNA was eluted in 50 μl RNase-free water, and 2 μl were used to quantify RNA concentration and for quality analysis (Agilent 2100 bioanalyzer (Agilent Technologies, Palo Alto, CA, USA). Immediately after extraction, total RNA was cooled to − 80 °C until further use.

### RNA-seq analysis

RNA-seq libraries were prepared from three grouped gonads from three E11 and E12 males and females. The cDNA libraries were sequenced using a HiSeq2500 v4 chemistry system at the Centre of Genomic Regulation (Barcelona, Spain). Sequencing yielded an average of 90 million125 bp paired-end sequences per sample. The quality of the samples was assessed with FastQC (http://www.bioinformatics.babraham.ac.uk/projects/fastqc/). Sequences were then trimmed using Trimmomatic software v0.36 [62].

### Differential gene expression analysis

Each sample was aligned against the reference genome (Mmu10/GrCm38.p6) using STAR software v2.5 [63]. Pure read counts were extracted from the alignment files using HTSeq-count v0.80 [64]. Subsequent differential gene expression analyses were performed independently using DESeq2 v.1.20.0 [65] and edgeR 3.22.3 [66, 67], keeping solely genes detected as differentially expressed (adjusted *p*-value < 0.01) by both programmes to improve the reliability of the findings. Gene Ontology enrichment analysis was performed with the David Gene Functional Classification Tool [68]. Terms with a p-value below 0.01 were taken to show significant enrichment.

### Gene expression analysis by quantitative RT-PCR

Quantitative RT-PCR was conducted following standard protocols [69]. RNA was converted to cDNA using a reverse transcription kit (Applied Biosystems, CA, USA). To prime the RT reaction and synthesize cDNA, poly(T) 35 primer, random primers and Moloney murine leukemia virus (MMLV) reverse transcriptase enzyme were used in a total volume of 40 mL [70]. All qPCR reactions were carried out in duplicate in the Rotorgene 6000 Real Time Cycler™ (Corbett Research, Sydney, Australia) using a PCR mix (GoTaq® qPCR Master Mix, Promega Corporation, MA, USA) containing the specific primers selected for the genes (see Additional file 25).

### Gene cluster analysis

Gene clustering was examined using Cluster Locator (http://clusterlocator.bnd.edu.uy/). A cluster was defined as the maximal set of genes where the gap between adjacent genes is never larger than a given max-gap set. First, we compared the percentage clustering obtained with our list of genes to the percentage obtained with random clustering (1000 lists of the same size as the analyzed list with genes randomly picked out from the same reference genome) with different max-gap settings (1–10, 15, 20, 25, 50, 75 and 100). With this information, we set the max-gap at 8 to analyze the clustering of the different lists of interest (genes upregulated in E11 and E12 males or females). The clusters obtained consisting of 5 or more genes were represented using Phenogram (http://visualization.ritchielab.org/phenograms/document). To study how these gene clusters fit with previously described HSBs on mammalian chromosomes, we obtained the genes included within the HSBs regions described by Larkin et al. (2009) [16]. After that, we calculated the percentage of genes from our lists of clustered genes (up regulated in male or female, at E11 and E12) included in the list of genes in the HSBs. We also generated 1000 random lists of the same size than the analyzed lists with genes selected randomly from the reference genome and we evaluated the percentage of these genes included in HSBs. We then compared the percentage of genes matching with HSBs obtained in our lists with the average percentage obtained with the 1000 random lists. To see if this difference was significative, we performed Chi-square test (2-sample test for equality of proportions without continuity correction).

### Differential isoform expression analysis

Transcript counts were first obtained using Salmon software v.0.11.2 [18]. Differential isoform usage was then analyzed using edgeR [66, 67], considering an isoform as differentially expressed when the adjusted *p*-value in the comparison between samples was below 0.01.

### Differential splicing analysis

Differential splicing was assessed using the vast-tools software package v2.0.1 (https://github.com/vastgroup/vast-tools), normalizing the distribution of each event by the frequency of that event in the mouse transcriptome (Mmu10). Differentially spliced events in males vs females (at E11 and E12) were determined by calculating the difference in their average inclusion levels (ΔPSI), considering only those ΔPSI that were higher than 0.1. Events were classified as: exon skipping (ES), alternative 3’splice site (3’ss), alternative 5′ splice site (5’ss), intron retention (IR) and microexon (MIC). Intron retention events, which correspond to U12 events, were then identified comparing our results with mouse U12 events obtained from U12db (http://genome.crg.es/cgi-bin/u12db/u12db.cgi) [39].

### Statistical analysis

Basic statistical analysis and graph construction were conducted using Microsoft Excel and R. Differences in qPCR gene transcription were analyzed by one-way repeated-measures analysis of variance (ANOVA) and significance determined using the Holm-Sidak post hoc test. Significance was set at *P* ≤ 0.05.

## Additional files

Additional file 1: (A) Summary of RNA sample, RNA-seq data, and number of genes and transcripts detected. (B) Summary of differentially expressed genes (DEGs) detected (*P* < 0.01). (C) Summary of differentially expressed isoforms (DEIs) detected (P < 0.01). (D) Comparison between DEGs and DEIs detected. (E) Summary of alternative splicing events detected. (DOCX 26 kb)

Additional file 2: Quantification and differential expression analysis at the gene and isoform level. Normalized FPKM of the expression values are provided in Tab (A) for each gene and Tab (C) for each isoform with detectable expression (expected counts > 0) along with GRCm38.p6 annotations. A graphing tool for easy visualization of gene expression (Tab. B) or isoform (Tab. D) in E11 and E12 XX and XY fetal gonads. To generate an expression graph for any chosen gene, the gene symbol is typed or pasted into cell A2 of Tab (B) or the transcript ID into cell A2 of Tab (D). (XLSX 27004 kb)

Additional file 3: Differentially expressed genes in males vs females at E11 (A), E12 (B) and at E11 vs E12 in males (C) and females (D). Sex biased genes are represented in (E). (XLSX 3483 kb)

Additional file 4: Gene Ontology analysis of the top 20 significantly enriched biological functions of genes upregulated in the genital ridges of E11 males (A) and females (B) and E12 males (C) and females (D). (XLSX 334 kb)

Additional file 5: Heat map of differentially expressed genes in the gonads of males and females at E11 that are downregulated at E12 in their respective sex (A: female and B: male analysis) and upregulated in the opposite sex. (PPTX 119 kb)

Additional file 6: List of genes upregulated in males or females at E11 and E12 genital ridges (B, E, H, K), upregulated in E11 or E12 in male or female genital ridges (A, D, G, J), and specific genes for E11 and E12 male or female genital ridges (C, F, I, L). (XLS 2301 kb)

Additional file 7: List of genes (A) and isoforms (B) showing male- or female biased expression. (XLSX 481 kb)

Additional file 8: Chromosome distribution profile for clusters of differentially expressed genes and comparison with homologous synteny blocks (HSBs). Distribution of clusters identified with Cluster Locator [15] upregulated in the gonads of E11 females (**A**), E12 females (**B**) E11 males (**C**) and E12 males (**D**). Only clusters > 5 genes are represented in the figures. Genes also present in HSBs are highlighted inside a black box. (XLSX 588 kb)

Additional file 9: Differentially expressed isoforms **(**DEI) in males vs females at E11 (A), at E12 (B) and at E11 vs E12 in males (C) and females (D). (XLSX 2931 kb)

Additional file 10: Relative contributions of transcriptional activation and repression to sexually dimorphic DEGs and DEIs for E11 vs E12. Venn diagrams comparing genes manifesting biased expression in one sex, those upregulated in the relevant sex, and those downregulated in the opposite sex. (XLSX 123 kb)

Additional file 11: Gene Ontology analysis of the top 20 significantly enriched biological functions of isoforms upregulated in the genital ridges of E11 males (A) and females (B) and E12 males (C) and females (D). (XLSX 316 kb)

Additional file 12: List of isoforms upregulated in males or females at E11 and E12 genital ridges (B, E, H, K), upregulated in E11 or E12 in male or female genital ridges (A, D, G, J), and specific isoforms for E11 and E12 male or female genital ridges (C, F, I, L). (XLSX 1224 kb)

Additional file 13: (A) List of up- or down regulated genes in male and female genital ridges during the time course E11 to E12. (B) List of up- or down regulated isoforms in male and female genital ridges during the time course E11 to E12. (XLSX 429 kb)

Additional file 14: List of upregulated alternative splicing (AS) events in E11 male genital ridges. AS categories: 3SS, alternative 3′ splice sites; A5SS, alternative 5′ splice sites; ES, exon skipping; IR, retained introns; U2&U12inU2, U2 IR plus U2 intron in U12-containing genes. (XLSX 149 kb)

Additional file 15: List of upregulated alternative splicing (AS) events in E12 male genital ridges. AS categories: 3SS, alternative 3′ splice sites; A5SS, alternative 5′ splice sites; ES, exon skipping; IR, retained introns; U2&U12inU2, U2 IR plus U2 intron in U12-containing genes. (XLSX 252 kb)

Additional file 16: List of upregulated alternative splicing (AS) events in E11 female genital ridges. AS categories: 3SS, alternative 3′ splice sites; A5SS, alternative 5′ splice sites; ES, exon skipping; IR, retained introns; U2&U12inU2, U2 IR plus U2 intron in U12-containing genes. (XLSX 478 kb)

Additional file 17: List of upregulated alternative splicing (AS) events in E12 female genital ridges. AS categories: 3SS, alternative 3′ splice sites; A5SS, alternative 5′ splice sites; ES, exon skipping; IR, retained introns; U2&U12inU2, U2 IR plus U2 intron in U12-containing genes. (XLSX 371 kb)

Additional file 18: List of genes showing different alternative splicing (AS) events in males vs females at E11 (A) and E12 (B). (C) Search tool for genes showing different AS events in males vs females at E11 and E12. (D) Functional annotation analysis (DAVID) of genes showing different AS events in males vs female at E11 and E12. (XLSX 77 kb)

Additional file 19: Comparison of DEGs with the data of Zhao et al. (A) List of DEGs found in our study. (B) List of DEGs found by Zhao et al. (C) Common differentially expressed genes in E11 vs E12 males. (D) Common differentially expressed genes in E11 vs E12 females. (XLSX 388 kb)

Additional file 20: Comparison between differentially expressed genes detected in the present study and previous RNA-seq data. Venn diagrams comparing the present data set with those of Zhao et al. [32] are shown. DEGs overexpressed in E11 (A) and E12 (B) males and E11 (C) and E12 (D) females. (PDF 143 kb)

Additional file 21: Comparison of differentially expressed isoforms detected in the present study and previous RNA-seq data. Venn diagrams comparing the present data set with those of Zhao et al. [32] are shown. DEIs overexpressed in E11 (A) and E12 (B) males and E11 (C) and E12 (D) females. (PDF 110 kb)

Additional file 22: Comparison of DEIs with the data of Zhao et al. and Jameson et al. (A) List of DEIs found in our study. (B) List of DEIs found by Zhao et al. Common differentially expressed isoforms in E11 vs E12 males to those detected by Zhao et al. (C) and by Jameson et al. (E). Common differentially expressed isoforms in E11 vs E12 females to those detected by Zhao et al. (D) and by Jameson et al. (F). (XLSX 342 kb)

Additional file 23: List of events common to DEIs and AS in male and female E11 and E12 genital ridges. (XLSX 1078 kb)

Additional file 24: List of events common to DEGs and DEIs in male and female E11 and E12 genital ridges. (XLSX 390 kb)

Additional file 25: Sequences of the primers used in this study. (XLSX 11 kb)

## Abbreviations

3SS: 3′ Alternative spliced site
5ss: 5′ Alternative spliced site
AS: Alternative splicing
Chr: Chromosome
DEG: Differentially expressed gene
DEI: Differentially expressed isoform
DSD: Disorder of sexual development
E: Embryonic day
ES: Exon skipping
GO: Gene ontology
GSD: Gonadal sex determination
HSB: Homologous synteny block
IR: Intron retention
MIC: Micro-exon
