# Supplementary material for: Differential isoform expression and alternative splicing in sex determination in mice
Source: BMC Genomics. 2019 Mar 12;20:202. doi: 10.1186/s12864-019-5572-x (PMC6419433; doi:10.1186/s12864-019-5572-x)
Supplement: Supplementary file 1 — (A) Summary of RNA sample, RNA-seq data, and number of genes and transcripts detected. (B) Summary of differentially expressed genes (DEGs) detected (P < 0.01). (C) Summary of differentially expressed isoforms (DEIs) detected (P < 0.01). (D) Comparison between DEGs and DEIs detected. (E) Summary of alternative splicing events detected. (DOCX 26 kb) [file 12864_2019_5572_MOESM1_ESM.docx]

| Sample | Total aligned reads | Number of genes detected | Number of transcripts detected |
| --- | --- | --- | --- |
| M11-1 | 86,350,882 | 32,749 | 98,851 |
| M11-2 | 89,318,911 | 34,719 | 102,293 |
| F11-1 | 87,210,597 | 34,708 | 99,188 |
| F11-2 | 87,902,105 | 37,675 | 105,881 |
| F11-3 | 85,351,537 | 37,883 | 105,077 |
| M12-1 | 87,093,688 | 35,437 | 103,501 |
| M12-2 | 88,200,720 | 36,006 | 104,092 |
| F12-1 | 87,140,305 | 34,793 | 102,086 |
| F12-2 | 90,129,335 | 35,673 | 102,325 |

**Additional Table 1A**. Summary of RNA-Seq data

For the analysis, we used the last version of the mouse genome (mmu10/GRCm38.p6). Number of genes with nucleotide sequence data in the reference genome: 49,142.
Number of transcripts in the reference transcriptome: 132,222.

**Additional Table 1B**. Summary of DEGs detected (P<0.01)

|  | MvsF E11 | | | MvsF E12 | | | Female E11vsE12 | | | | Male E11vsE12 | | | |  |
| --- | --- | --- | --- | --- | --- | --- | --- | --- | --- | --- | --- | --- | --- | --- | --- |
|  | Male Up | Female Up | Total | Male Up | Female Up | Total | | E11 Up | E12 Up | Total | | E11 Up | E12 Up | Total | |
| DESeq2 | 1104 | 924 | 2028 | 1022 | 950 | 1972 | | 3145 | 4184 | 7329 | | 2273 | 2275 | 4548 | |
| edgeR | 739 | 847 | 1586 | 1166 | 1212 | 2378 | | 3174 | 5264 | 8438 | | 2225 | 1927 | 4152 | |
| Both test | 697 | 531 | 1228 | 957 | 892 | 1849 | | 2882 | 4184 | 7066 | | 1897 | 1685 | 3582 | |

**Additional Table 1C**. Summary of DEIs detected (P<0.01)

|  | MvsF E11 | | | MvsF E12 | | | Female E11vsE12 | | | Male E11vsE12 | | |
| --- | --- | --- | --- | --- | --- | --- | --- | --- | --- | --- | --- | --- |
|  | Male Up | Female Up | Total | Male Up | Female Up | Total | E11 Up | E12 Up | Total | E11 Up | E12 Up | Total |
| Coding | 283 | 363 | 646 | 767 | 570 | 1337 | 1998 | 2936 | 4934 | 1773 | 1528 | 3301 |
| Non coding | 162 | 256 | 418 | 210 | 155 | 365 | 550 | 862 | 1412 | 509 | 505 | 1014 |
| All | 445 | 619 | 1064 | 977 | 725 | 1702 | 2548 | 3798 | 6346 | 2282 | 2033 | 4315 |

**Additional Table 1D**. Comparison between DEGs and DEIs detected

|  | MvsF E11 | | | MvsF E12 | | | Female E11vsE12 | | | Male E11vsE12 | | |
| --- | --- | --- | --- | --- | --- | --- | --- | --- | --- | --- | --- | --- |
|  | Male Up | Female Up | Total | Male Up | Female Up | Total | E11 Up | E12 Up | Total | E11 Up | E12 Up | Total |
| DEG | 697 | 531 | 1228 | 957 | 892 | 1849 | 2882 | 4184 | 7066 | 1897 | 1685 | 3582 |
| DEI | 445 | 619 | 1064 | 977 | 725 | 1702 | 2548 | 3798 | 6346 | 2282 | 2033 | 4315 |
| Common events | 104 | 96 | 200 | 676 | 495 | 1171 | 1988 | 2948 | 4944 | 1590 | 1333 | 2923 |
| % DEI commons | 23.37 | 15.67 | 18.80 | 69.19 | 68.28 | 68.80 | 78.02 | 77.80 | 77.62 | 69.68 | 65.57 | 67.74 |

**Additional Table 1E**. Summary of alternative splicing events detected

|  | Up-regulated in female | | Up-regulated in male | |
| --- | --- | --- | --- | --- |
|  | E11 | E12 | E11 | E12 |
| 3SS | 93 | 134 | 86 | 123 |
| 5SS | 125 | 144 | 97 | 130 |
| ES | 180 | 334 | 79 | 185 |
| MIC | 3 | 4 | 1 | 2 |
| IR (U12) | 843 (70) | 376 (22) | 111 (14) | 218 (29) |
| Total | 1244 | 992 | 374 | 658 |

U12: gene with U12 introns, with alternative splicing events in U12 or U2 introns.
