# Supplementary material for: Differential isoform expression and alternative splicing in sex determination in mice
Source: BMC Genomics. 2019 Mar 12;20:202. doi: 10.1186/s12864-019-5572-x (PMC6419433; doi:10.1186/s12864-019-5572-x)
Supplement: Supplementary file 5 — Heat map of differentially expressed genes in the gonads of males and females at E11 that are downregulated at E12 in their respective sex (A: female and B: male analysis) and upregulated in the opposite sex. (PPTX 119 kb) [file 12864_2019_5572_MOESM5_ESM.pptx]

## Slide 1
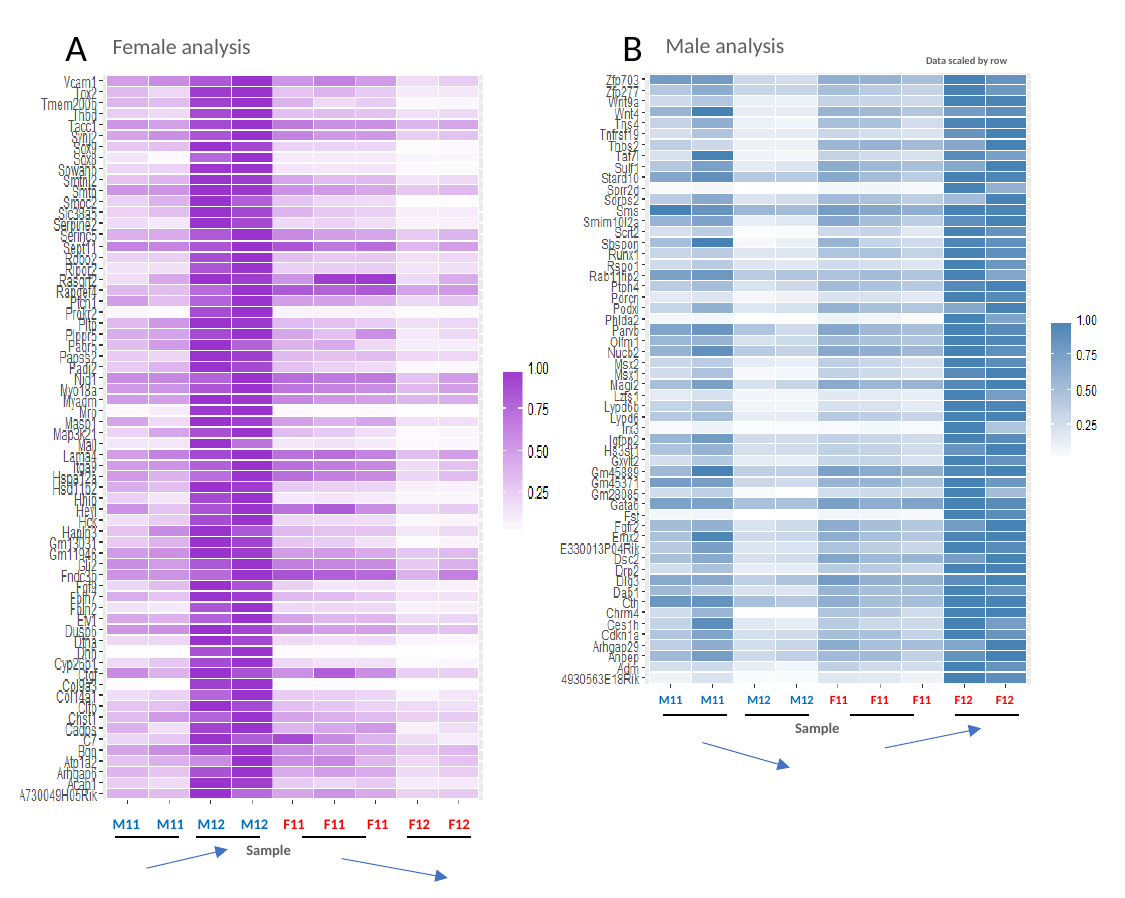

A
B
Male analysis
Female analysis
Data scaled by row
Genes
Genes
F12
F12
F11
F11
M12
F11
M12
M11
M11
Sample
Sample
F12
F12
F11
F11
M12
F11
M12
M11
M11
Sample
