# Supplementary material for: Differential isoform expression and alternative splicing in sex determination in mice
Source: BMC Genomics. 2019 Mar 12;20:202. doi: 10.1186/s12864-019-5572-x (PMC6419433; doi:10.1186/s12864-019-5572-x)

Overexpressed transcripts in male

Comparison with Zhao *et al*, 2018

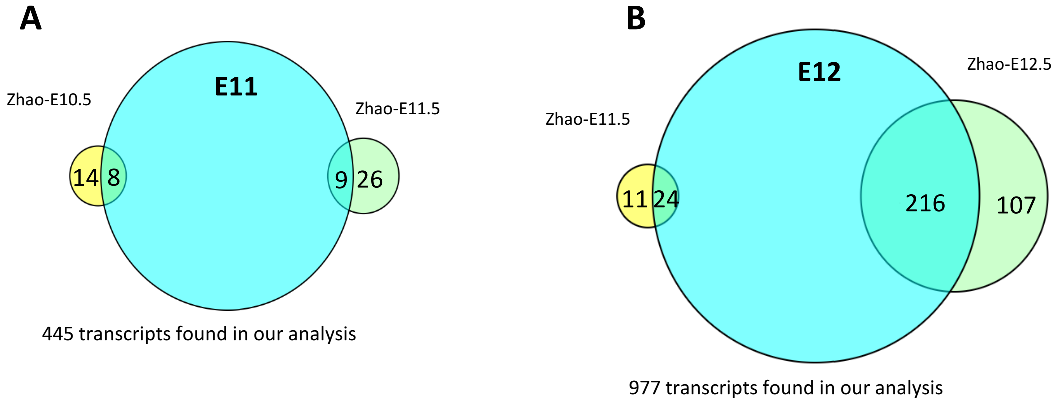

Overexpressed transcripts in female

Comparison with Zhao *et al*, 2018

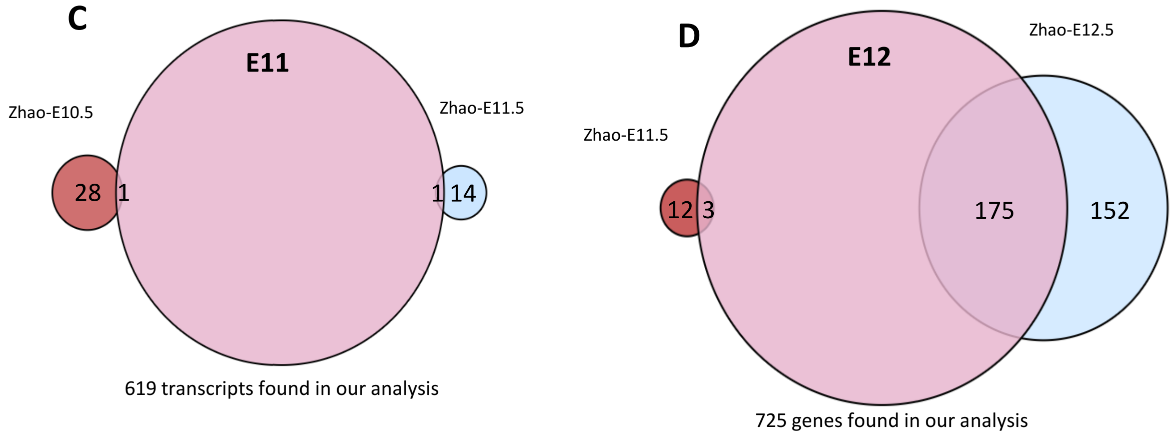

Supplement: Supplementary file 21 — Comparison of differentially expressed isoforms detected in the present study and previous RNA-seq data. Venn diagrams comparing the present data set with those of Zhao et al. [32] are shown. DEIs overexpressed in E11 (A) and E12 (B) males and E11 (C) and E12 (D) females. (PDF 110 kb) [file 12864_2019_5572_MOESM21_ESM.pdf]
